# Chromatin Accessibility Regulates Gene Expression and Correlates With Tumor-Infiltrating Immune Cells in Gastric Adenocarcinoma

**DOI:** 10.3389/fonc.2020.609940

**Published:** 2021-01-05

**Authors:** Chenshen Huang, Runzhi Huang, Hong Chen, Zhizhan Ni, Qi Huang, Zongqiang Huang, Bujun Ge

**Affiliations:** ^1^ Department of General Surgery, Tongji Hospital, Tongji University School of Medicine, Shanghai, China; ^2^ Department of Orthopedics, The First Affiliated Hospital of Zhengzhou University, Zhengzhou, China; ^3^ Division of Spine, Department of Orthopedics, Tongji Hospital affiliated to Tongji University School of Medicine, Shanghai, China; ^4^ Center for Difficult and Complicated Abdominal Surgery, Shanghai Tenth People’s Hospital, Tongji University School of Medicine, Shanghai, China

**Keywords:** IL18BP, chromatin accessibility, CD4+ T cells, gastric adenocarcinoma, ATAC-seq, tumor-infiltrating immune cells

## Abstract

**Background:**

We explored key molecules affecting the prognosis of gastric adenocarcinoma (STAD) using co-analysis of chromatin accessibility (ATAC-seq), mRNA expression (RNA-seq), and overall survival.

**Methods:**

We used the assay for transposase-accessible chromatin using sequencing (ATAC-seq) profiles to identify genes with chromatin accessibilities in their promoter regions. The RNA-seq profiles were processed for differentially expressed genes (DEGs) at mRNA level. The DEGs with chromatin accessibilities in promoter regions were further filtered using the Pearson correlation with TP53 expression. After co-analysis, genes were identified for the prognostic value using Kaplan–Meier method, followed by Pearson correlation analysis with significant pathways. For verification, we acquired clinical samples for qPCR and immunohistochemistry (IHC). Multidimensional database validations were performed to prevent the bias introduced by the use of a single database.

**Results:**

We identified 11,557 DEGs and 57 genes with chromatin accessibilities. The co-analysis of ATAC-seq, RNA-seq, and clinical survival data revealed that interleukin-18 binding protein (IL18BP), with significant chromatin accessibility in its promoter region and differential mRNA expression, might be directly regulated by TP53 and influence STAD prognosis. Further, gene set variation analysis indicated that IL18BP may impact the survival of STAD patients in an immune-related manner. According to the CIBERSORT algorithm and Pearson correlation, the integration of IL18BP and CD4+ T memory cells may play an important role in the prognosis of STAD patients.

**Conclusion:**

IL18BP, regulated by TP53, may serve as a key molecule affecting STAD prognosis. And the mechanism is proposed to be the interaction between IL18BP and CD4+ T cells.

## Introduction

Gastric cancer, a malignant tumor type, is the fifth most common cancer, and the third leading cause of cancer deaths worldwide (1). Gastric adenocarcinoma (STAD) is the most common (approximately 95% of all cases) pathological subtype of gastric cancer (2). Currently, the prognosis of patients with advanced STAD remains poor. Therefore, a better understanding of the key molecules of STAD prognosis through integrated bioinformatics is warranted.

TP53 encodes the p53 protein, an important factor in cancer development, and is one of the most frequently mutated genes in STAD. Despite extensive study of the p53 network, an improved understanding of its diverse effects is still needed (3, 4). In addition, as a transcription factor (TF), mutant p53 had been proposed to be associated with chromatin states (3). Therefore, we inferred that p53 might affect STAD prognosis at the epigenetic level. So we acquired ATAC-seq profiles and explored the genes that might be directly regulated by p53.

Assay for transposase-accessible chromatin using sequencing (ATAC-seq) enables mapping of open chromatin sites, predict TF binding, and determine nucleosome position using Tn5 transposase (5–7), even in single cells (8, 9). ATAC-seq detects chromatin accessibility of related genes and indicates the regulatory mechanism. Open chromatin sites in promoter regions reflect the potential of TF binding. Therefore, a gene with chromatin accessibility in its promoter is more likely to be regulated by TF and expressed differentially at the mRNA level. Here, we explored significant and reproducible chromatin accessibilities in patients with STAD to identify a group of genes regulated by TP53. We followed this up with RNA-seq analysis, to verify the differential mRNA expression levels of these genes. The acquired TP53 ChIP-seq data also validated the direct regulation. This co-analysis approach, taking both epigenetics and transcriptomics into consideration, might be a more effective way to explore the potential targets for STAD treatment.

Tumor immunology is thought to impact the development of STAD, and tumor infiltrating lymphocytes (TILs) may be used as therapeutic targets or prognostic predictive markers (10–13). The “cell type identification by estimating relative subsets of RNA transcripts (CIBERSORT)” (14) has made it effective to quantitatively describe the fraction of TILs. Until now, the relationships between TP53 and TILs are still poorly studied.

Here, we used ATAC-seq, RNA-seq and CIBERSORT to explore the correlations among chromatin accessibility, gene expression and TILs. We found that IL18BP, regulated by TP53, had significant chromatin accessibilities in the promoter region and was differentially expressed in STAD. The expression level of IL18BP was correlated with the fraction of CD4+ T memory cells, both of which showed prognostic value for patients with STAD. Also, we constructed a STAD prognosis predicting nomogram based on significant TILs, identified using the LASSO and multi-cox regression models. We verified the above-mentioned results using qPCR, immunohistochemistry (IHC), and multidimensional databases.

## Materials and Methods

### Ethics

The ethics approval of our study was obtained from Shanghai Tongji Hospital (reference number 2018-LCYJ-005). The written consent from all the participants were obtained before surgery.

### Data Collection

The gene expression profiles of STAD and normal solid tissue samples were obtained from TCGA database (https://tcga-data.nci.nih.gov/tcga/). We acquired the HTseq-counts and fragments per kilobase per million (FPKM) profiles of 407 samples, comprising 375 STAD and 32 solid normal tissue samples. To minimize the impact of patient death due to non-tumor-related reasons, clinical demographic information of patients with a follow-up time of over 90 days was retrieved. The baseline information of STAD samples were summarized in **Table S1**. We also retrieved the ATAC-seq profiles of 21 STAD samples from TCGA database (https://tcga-data.nci.nih.gov/tcga/).

### Chromatin Accessibility Analysis in ATAC-seq

To explore the chromatin accessibilities, we first used the R package karyoploteR to display the peak regions over chromosomes for visualization. Peaks that could be mapped to the TSS regions were aligned to construct the tagMatrix by the R package ChIPseeker. The nearest TSS regions were selected for peak annotations. The annotation information was acquired from R (TxDb. Hsapiens. UCSC. hg38. knownGene). A Venn diagram and an upset plot were constructed to reveal the relationship between open chromatin and promoter regions.

### Differentially Expressed Genes (DEGs) in RNA-seq

To explore the STAD-specific gene expression, we used the edgeR method to identify differentially expression genes (DEGs) between STAD and normal solid tissue samples by false discovery rate (FDR) p-value and log2(Fold-change). FDR P < 0.05 and log2 (Fold-Change) >1.0 or <−1.0 were defined as either downregulated or upregulated, respectively.

### Correlation Between Gene Expression and KEGG Pathways

Gene set variation analysis (GSVA) (15) was performed for the expression of pathways in patients with STAD. KEGG pathways were identified using the GSEA database (https://www.gsea-msigdb.org/gsea/downloads.jsp). We carried out Pearson analysis for the co-analysis of gene expressions and KEGG pathways.

### Infiltrating Immune Cells

We used the CIBERSORT algorithm to estimate data on tumor-infiltrating immune cells. After the CIBERSORT processing, samples with p < 0.05 were considered eligible and used for subsequent studies. The normalized gene expression matrix was transformed into a corresponding infiltrating immune cell expression matrix by CIBERSORT, which used a deconvolution algorithm and Monte Carlo sampling to provide a reliable composition of infiltrating immune cells. Wilcoxon rank-test was used to screen for infiltrating immune cells with significant differences between tumor and normal solid tissue samples. Additionally, the multi-cox regression and Kaplan–Meier analyses were used to acquire the correlation between the immune cell fraction and the overall survival of gastric adenocarcinoma patients.

### Integrative Analysis of Chromatin Accessibility (ATAC-seq), Gene Expression (RNA-seq), Overall Survival (Kaplan-Meier), and TILs (CIBERSORT Algorithm)

To find the gene directly downstream of TP53, the DEGs included in the results of ATAC-seq analysis were filtered by the Pearson correlation analysis with TP53 mRNA expression and by the ChIP-seq data of p53 protein. After the filtration, genes were further identified for prognostic value using the Kaplan–Meier method. The optimal cutpoint was determined by the R package survminer (https://CRAN.R-project.org/package=survminer). This package used maximally selected rank statistics to determined the cutoffs. Then, the Pearson correlation analysis of these genes and TILs was performed.

### Prediction Nomogram

We used multi-cox regression and Kaplan–Meier survival analysis to determine the relationship between tumor infiltrating immune cells and the overall survival of patients with STAD. The prognostic value of immune cells was evaluated by survival analysis. All the significant immune cells were obtained from the multi-cox model. The Lasso regression was used to ensure that there was no overfitting in the model and the reduced multi-cox model of the significant markers was constructed. Finally, based on the multi-cox model, we constructed a nomogram to predict the prognosis of patients with STAD. Calibration curves validated the discrimination and accuracy of the prediction nomogram.

### qRT-PCR Validation

Fresh, clinical samples of STAD and paired normal solid tissues were acquired from 30 patients from the Tongji Hospital, Shanghai, China. Total RNA was extracted with Trizol reagent (Magen, R4801-01) in accordance with the manufacturer’s instructions. Quantitative PCR was carried out using SyberGreen(Yeasen, 11200ES03), cDNA and the human-IL18BP primers (Forward: ATGAGACACAACTGGACACCA, Reverse: GCCAGGTCACTTCCAATGC). The amplification program was as follows: 95°C for 10 min; 40 cycles of 95°C for 10 s, 58°C for 20 s, and 72°C for 15 s; melting curve from 60 to 95°C, increasing in increments of 0.5°C every 5 s. Actin served as an internal control. The experiment was repeated three times and every sample in each group was detected in triplicate.

### Immunohistochemistry (IHC) Validation

IHC was performed in 12 paraffin-embedded gastric adenocarcinoma samples. Following overnight incubation at 4°C with the primary antibody anti-IL18BP (abcam, ab52914, 1:400), slides were washed three times and subsequently incubated with the secondary antibody for 1 h at room temperature. We used DAB (Servicebio, G1211) to stain the slides. We used hematoxylin for counterstaining of the nuclei. All slides were processed simultaneously with the same conditions. The percentage of positive tumors were further examined and counted by the IHC Profiler in ImageJ software (16).

### Multiple Database Validation

To minimize the bias and further validate our hypothesis, multidimensional validation was performed using the following online databases: Gene Expression Omnibus (GEO)(ID: GSE15459) (17), TIMER (http://timer.comp-genomics.org/) (18–20), GEPIA (http://gepia.cancer-pku.cn/) (21) and Kmplot (https://kmplot.com/analysis/).

### Statistical Analysis

All analyses were implemented in R version 3.5.1 software (Institute for Statistics and Mathematics, Vienna, Austria www.r-project.org). Statistical significance was set at p < 0.05.

## Results

### Identification of Accessible Chromatin in STADs Using ATAC-seq Analysis

**Figure 1:**
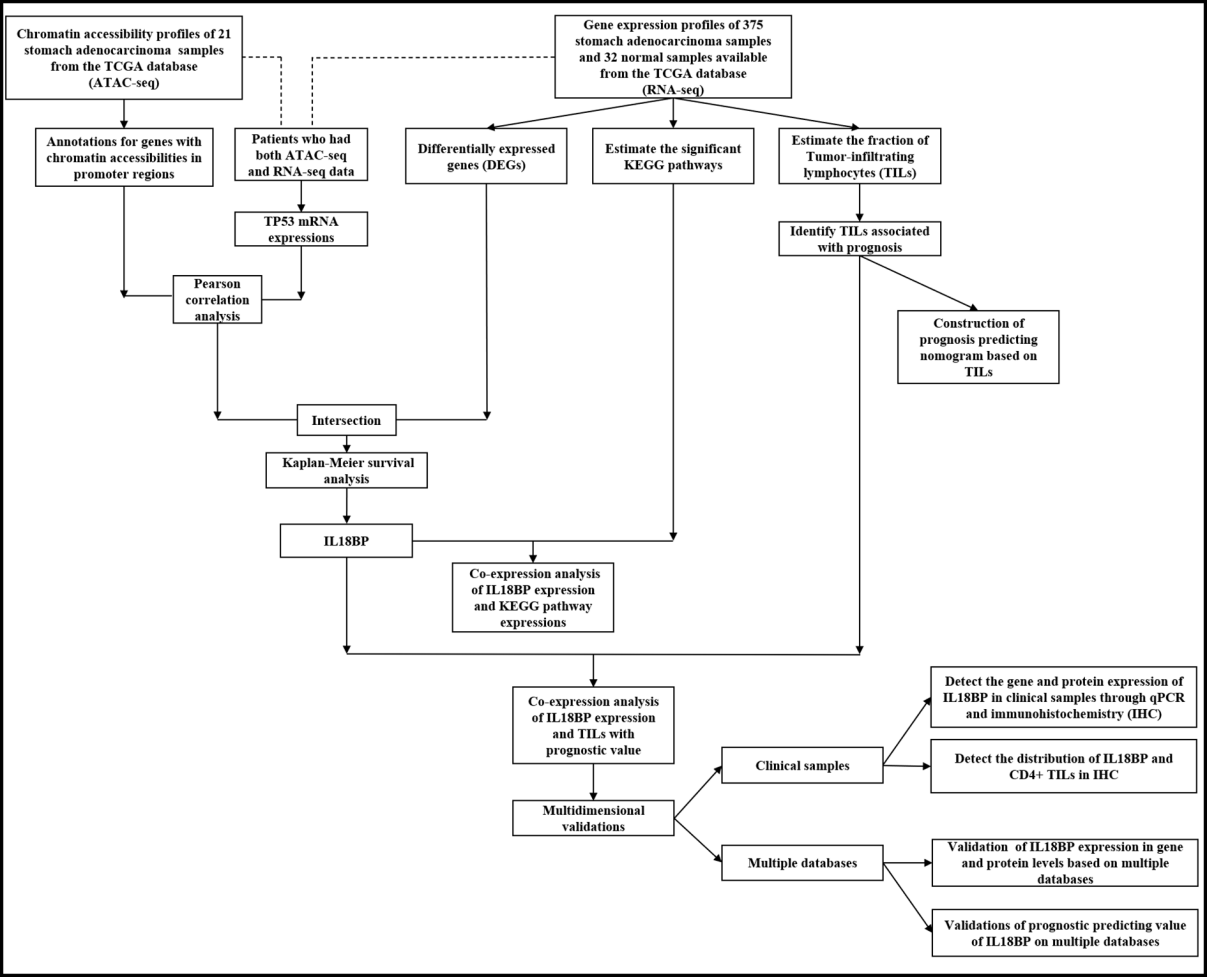
An overview of the workflow in this study.

**Figure 2:**
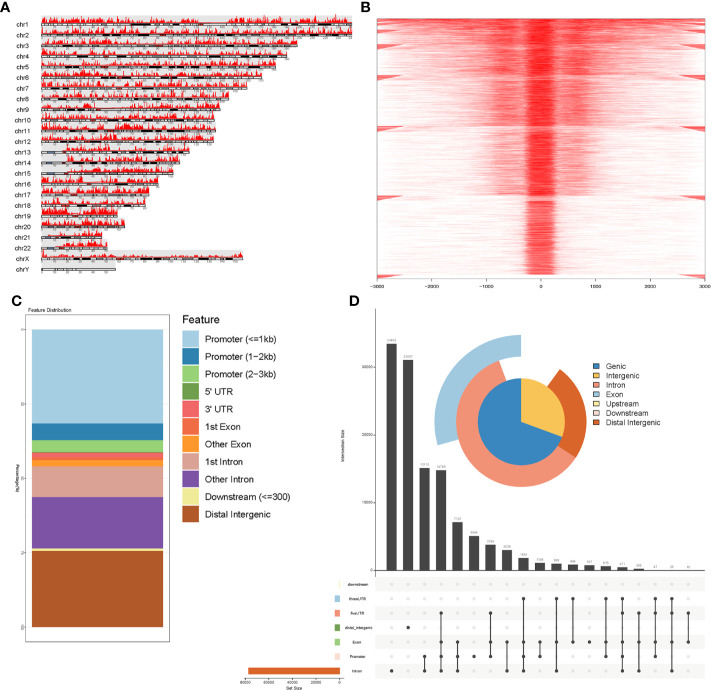
Identification of accessible chromatin in STADs using ATAC-seq analysis. **(A)** Peak calling function was performed to identify open chromatin regions. Visualization of peak regions over chromosomes revealed that chromatin accessibilities were widely present over the whole genome. **(B)** Peaks that could be mapped to the TSS regions were aligned to construct a tagMatrix. The majority of open chromatin regions were located around the TSS regions. **(C)** Annotation of chromatin accessibilities to the nearest genes indicated that most were located in the promoter regions. **(D)** Venn pie diagram and an upset plot indicated that most open chromatin regions were located in promoter and intronic regions.

An overview of our workflow is presented in **Figure 1**. The chromatin accessibility landscape of patients with STAD was derived from the ATAC-seq profile of 21 tumor samples from TCGA database. We identified 57 reproducible genes (**Table S2**) with accessible chromatin across samples. **Figure 2A** shows the distribution of accessibilities among chromosomes that were widely present over the whole genome. These accessible areas were found within the transcription start site (TSS) regions. Most open chromatin regions were located in promoter regions (**Figures 2B–C**). The upset plot and Venn pie diagram (**Figure 2D**) show the chromatin accessibility distributions with regulatory elements and revealed that chromatin accessibilities were mainly located in the promoter and intron regions.

### Identification of Prognostic Biomarker IL18BP

**Figure 3:**
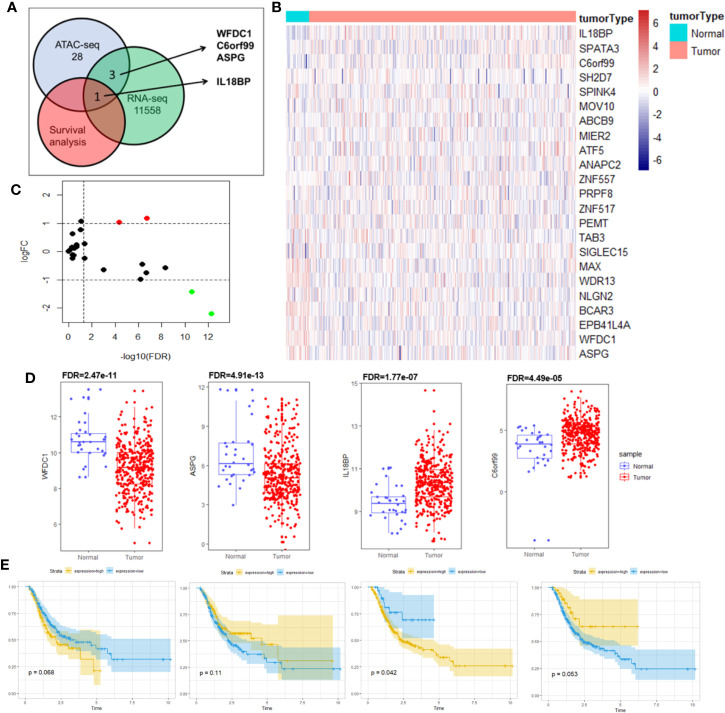
Identification of prognostic biomarker IL18BP by co-analysis of RNA-seq, ATAC-seq and overall survival. **(A)** Schematic for co-analysis of RNA-seq, ATAC-seq and Kaplan–Meier survival analysis in STAD. Four genes (WFDC1, C6orf99, IL18BP, and ASPG) were identified in the co-analysis of RNA-seq and ATAC-seq, and only IL18BP was found promising based on the Kaplan–Meier analysis. **(B)** Heatmap for mRNA expression of the 28 genes with chromatin accessibilities in its promoter regions in ATAC-seq analysis. Data of the mRNA expression were collected from TCGA database. **(C)** Differentially expressed genes were identified in analysis of RNA-seq of STAD (log (fold-change) >1.0 or log(fold-change) <−1.0; FDR < 0.05). The volcano plot revealed that among the 28 identified genes in ATAC-seq, four genes (WFDC1, C6orf99, IL18BP, and ASPG) were differentially expressed at the mRNA level. **(D)** The mRNA expression levels of WFDC1, C6orf99, IL18BP, and ASPG in **(B)**. WFDC1 and ASPG were downregulated in STAD tissues, whereas IL18BP and C6orf99 were upregulated. **(E)** Kaplan-Meier survival analysis revealed that only IL18BP showed significant prognosis value in patients with STAD (p < 0.05). The patients with higher IL18BP expression displayed poorer prognosis.

As transcription factors regulate gene expressions and their binding sites are located around the promoter, we searched for genes that possessed chromatin accessibilities in these regions. We performed a co-analysis of RNA-seq and ATAC-seq to search for the genes that had chromatin accessibilities in their promoter regions and were differentially expressed at the mRNA level in STAD. These genes were further filtered using the survival analysis to identify prognostic biomarkers. **Figure 3A** visualized our above-mentioned screening strategy.

We first acquired RNA-seq data from TCGA. **Table S1** describes the baseline characteristics of STAD samples obtained from TCGA database. Using log (fold-change) >1.0 or log(fold-change) <−1.0 and FDR < 0.05 as cutoffs, gene expression profiles of 375 STAD samples and 32 normal solid tissues were analyzed. We identified 11,557 differentially expressed protein-coding genes from RNA-seq analysis, including 3,636 and 7,921 downregulated and upregulated genes, respectively.

Of the 57 reproducible genes from the ATAC-seq analysis, 28 genes (**Table S2**) showed chromatin accessibilities in their promoter regions. We constructed a heatmap (**Figure 3B**) and volcano plot **(** **Figure 3C**) to show the mRNA expressions of these 28 genes. Among them, four (WFDC1, C6orf99, IL18BP, ASPG) were found to be differentially expressed at the mRNA level (**Figure 3D**) and were selected for further survival analysis.

Of the four genes, only IL18BP showed prognostic value in patients with STAD, demonstrated by the Kaplan–Meier analysis (p = 0.042; **Figure 3E**). Collectively, we identified IL18BP as a prognosis-related gene in patients with STAD. It was differentially expressed at the mRNA level in STAD samples and showed reproducible chromatin accessibilities in its promoter region.

### Verification of IL18BP Expression in STADs

**Figure 4:**
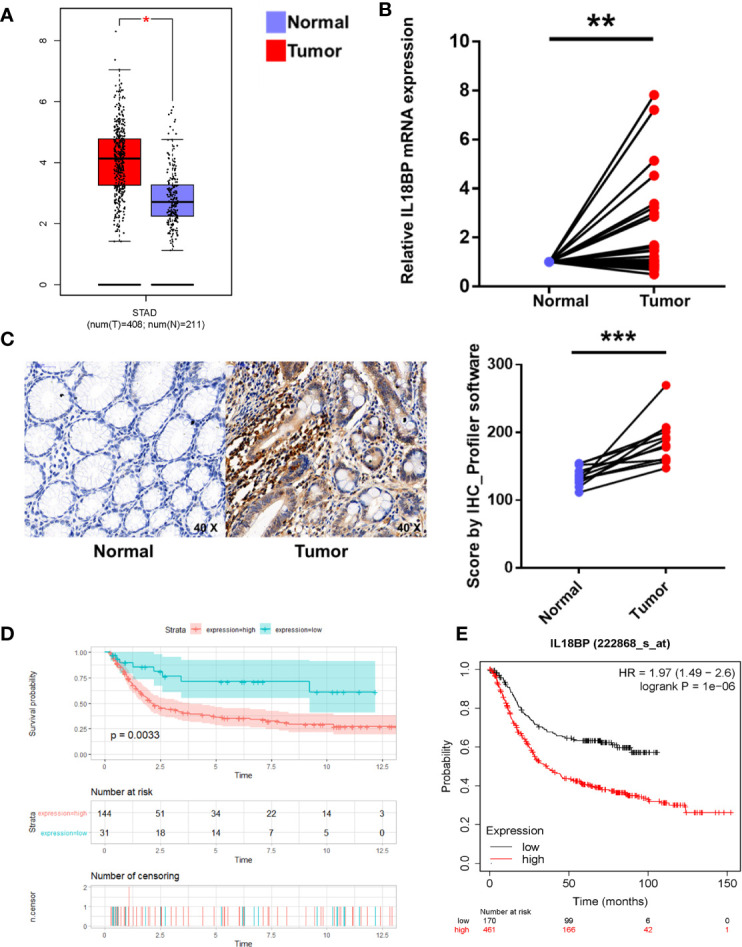
Multiple verifications of IL18BP expression in STAD. **(A)** STAD (n = 408) and normal (n = 211) tissues from the GEPIA database verified that IL18BP was significantly upregulated in STAD (p < 0.05). **(B)** A total of 30 fresh STAD and paired normal tissues were collected from Tongji Hospital, Shanghai, China. IL18BP was significantly upregulated in STAD tissues (p < 0.05), as shown in qPCR analysis. **(C)** IL18BP expression was significantly upregulated in STAD samples at the protein level, as shown by IHC (n = 12 per group). **(D)** IL18BP was a prognostic risk factor in patients with STAD (p = 0.003) according to the Kaplan-Meier analysis of GSE15459 from GEO database. **(E)** IL18BP was a prognostic risk factor in patients with STAD (log rank P =1e-06) from KMPLOT database.

To prevent the bias caused by bioinformatic analyses, we performed qPCR analysis of STAD tumor and paired normal tissues from 30 patients with STAD from Tongji Hospital. The qPCR findings were consistent with our bioinformatic findings that the IL18BP expression was significantly upregulated in gastric adenocarcinoma tissues (p = 0.0072, **Figure 4B**). To further study the IL18BP protein expression, we performed immunohistochemistry staining of 12 STAD tumor samples and normal solid tissues. The upregulation of IL18BP was also identified by immunohistochemistry (p = 0.0003, **Figure 4C**).

Additionally, to prevent any bias we used a multi-database approach to validate both gene and protein expression of IL18BP in STAD and normal tissues. The analysis of GEPIA dataset (http://gepia.cancer-pku.cn/) revealed that IL18BP was highly expressed in gastric adenocarcinoma compared with that of the normal tissues (**Figure 4A**). We also confirmed the prognostic value of IL18BP in patients with STAD in GEO (https://www.ncbi.nlm.nih.gov/geo/query/acc.cgi?acc=GSE15459) and KMplot (http://www.kmplot.com/) databases. Of these two datasets, the optimal cutoffs were determined by the maximally selected rank statistics method. In the GEO dataset GSE15459, patients with high IL18BP levels in tumor samples tended to have poorer survival outcomes compared with those with lower IL18BP levels (**Figure 4D**). Similar results were obtained from the KMplot database (**Figure 4E**).

### Identifying Significant Pathways Correlated With IL18BP

**Figure 5:**
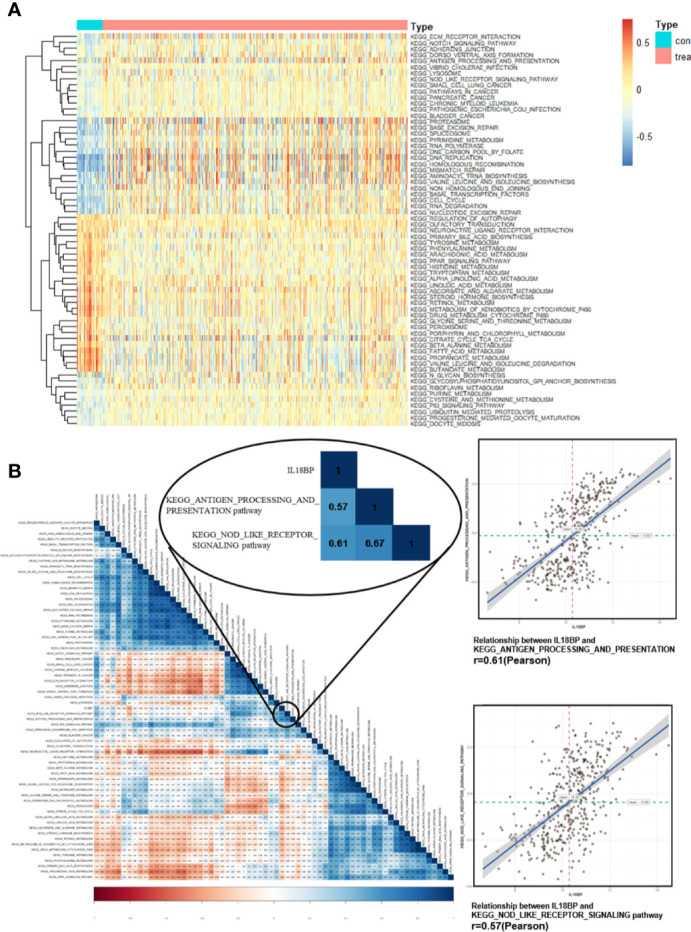
Identification of significant pathways correlated with IL18BP. **(A)** Heatmap displays the 65 significant KEGG pathways which are differentially expressed in STAD and normal tissues from TCGA database. **(B)** Correlation heatmap of the significant KEGG pathways and the expression of IL18BP. IL18BP was significantly correlated with the KEGG antigen processing and presentation [r = 0.61(Pearson), p < 0.001] and the nod-like receptor signaling [r = 0.57 (Pearson), p < 0.001] pathways.

To further identify the characteristic of IL18BP and related pathways in STAD, gene set variation analysis (GSVA) was performed for the expression level of KEGG pathways. The pathways which expressed differentially in STAD were displayed (**Figure 5A**). We also used Pearson analysis to explore the correlation between IL18BP and these significant pathways (**Figure 5B**). The largest correlation coefficient was used to decide the most significant pathway (p < 0.05). We found that the EGG_ANTIGEN_PROCESSING_AND_PRESENTATION (r = 0.61, p < 0.001) and the KEGG_NOD_LIKE_RECEPTOR_SIGNALING (r = 0.57, p < 0.001) pathways were most significantly correlated with IL18BP. Both pathways were also closely correlated with tumor immunology. Thus, we hypothesized that the prognostic predicting gene IL18BP might affect the survival of patients with STAD in an immunity-dependent way.

### CD4+ T Cells Could Predict STAD Prognosis

As IL18BP may influence the prognosis of patients with STAD in an immunity-related way, we aimed to find the subtypes of TILs that correlated with IL18BP expression and presented as good prognostic predictors.

**Figure 6:**
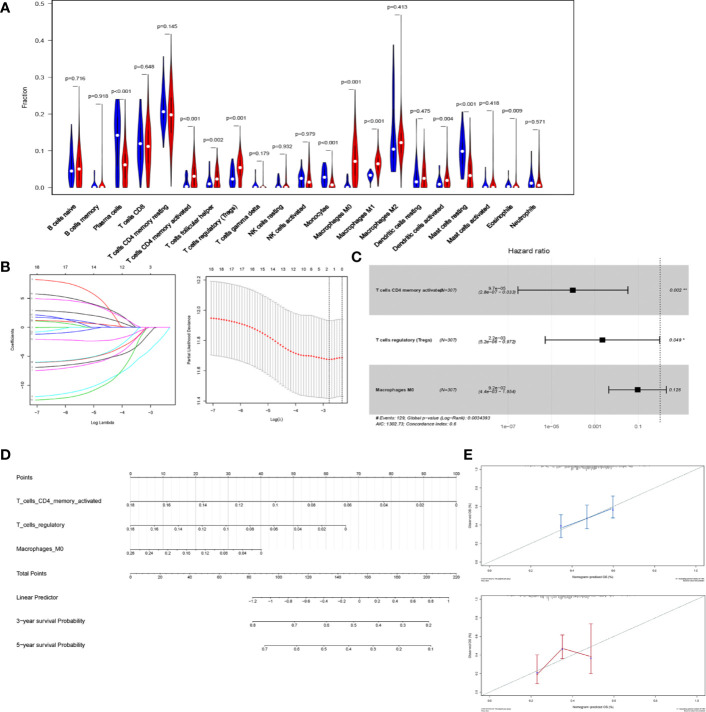
CD4+ T cells are a prognostic predicting marker in STAD. **(A)** Violin plot of the relative fraction of tumor-infiltrating immune cells, estimated by CIBERSORT in patients with STAD from TCGA database. **(B)** LASSO regression was performed to prevent the bias of overfitting. **(C)** Reduced multi-cox regression model based on the original cox regression model and LASSO regression. Activated CD4+ memory T cells (p = 0.002), Macrophage M0 (p = 0.126), and regulatory T cells (p=0.049) were identified to be potential markers predicting the prognosis of STAD. **(D)** Nomogram was constructed based on the reduced multi-cox regression model (C-index = 0.6). **(E)** The calibration curves of 3-year-survival (blue) and 5-year-survival (red) indicated the acceptable accuracy.

We first estimated the proportion of tumor-infiltrating immune lymphocytes (TILs) using CIBERSORT in STAD (**Figure 6A**). To indicate the prognostic value of immune cells, all infiltrating immune cells were processed using a reduced multi-cox regression model. In order to prevent overfitting, LASSO regression was utilized to screen for prognostic indicators of STAD (**Figure 6B**). Activated CD4+ memory T cells, Macrophages M0, and T cells regulatory (Tregs) were considered eligible in the results of LASSO regression and were further included in the reduced multi-cox regression model (C-index = 0.6, **Figure 6C**). The nomogram based on the multivariable model was also constructed (**Figure 6D**). From the reduced cox model and the nomogram, the expression level of activated CD4+ T memory cells, Macrophage M0, and Tregs were found to evaluate the prognosis effectively (**Figure 6E**).

Additionally, the reduced cox model in **Figure 6C** showed that Activated CD4+ memory T cells (P = 0.002) and Tregs (P = 0.049) had a significant P-value, indicating that they were more significant than Macrophages M0 (P = 0.126) in predicting the prognosis of STAD. Thus, the activated CD4+ memory T cells and Tregs were selected in further analysis.

### IL18BP Was Closely Related With Tumor Infiltrating CD4+ T Cells in STAD

**Figure 7:**
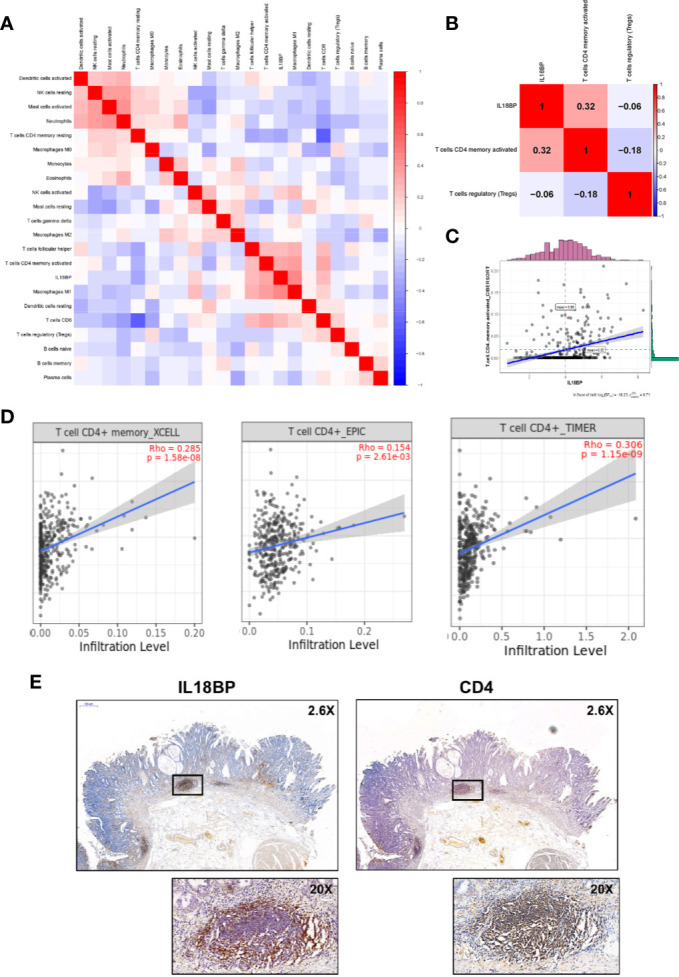
IL18BP was closely related to tumor infiltrating CD4+ T cells in STAD. **(A)** Pearson correlation analysis of the tumor-infiltrating immune cells and IL18BP. **(B)** The expression of IL18BP was highly correlated with activated CD4+ memory T cells [r = 0.32 (Pearson), p < 0.001] across the three immune cells identified in the reduced multi-cox model. **(C)** Dot plot revealed the correlation between IL18BP expression and activated CD4+ memory T cells [r = 0.31 (Pearson), p < 0.001]. **(D)** XCELL, EPIC, and TIMER algorithms confirmed a close correlation between CD4+ T cells and IL18BP. Data were acquired from TIMER database. **(E)** Immunohistochemistry demonstrated a close correlation between the distributions of IL18BP and CD4 at the protein level. The correlations were confirmed in the pathological sections of 12 patients with STAD.

**Figures 7A, B** show the significant co-expression between the tumor-infiltrating immune cells and IL18BP and the relationships between IL18BP and immune cells in the reduced cox model, respectively. IL18BP was significantly associated with activated CD4^+^ memory T cells (r = 0.32, p < 0.001, **Figure 7C**). We also explored the relationships among TP53, IL18BP, and immune cells (**Figures S1A–B**). Interestingly, TP53 was significantly associated with IL18BP (r = 0.39, p < 0.001). And the correlation between TP53 and activated CD4^+^ memory T cells was statistically significant but weak (r = 0.13, p = 0.01, **Figure S1B**). We inferred that TP53 might influence CD4^+^ memory T cells through IL18BP.

Additionally, the TIMER database (http://timer.comp-genomics.org/) was used for verification. We found that the fraction of CD4+ T cells was closely related to IL18BP expression in STAD tissues in the XCELL, EPIC, and TIMER algorithms (**Figure 7D**). The correlation between the distributions of IL18BP and CD4 was also confirmed with immunohistochemistry (**Figure 7E**). At the tissue level, the secreted protein IL18BP was enriched in the CD4 positive area. We hypothesized that IL18BP in STAD might play a role in the regulation of CD4+ T cells.

## Discussion

STAD is one of the most common tumors with a high mortality rate worldwide. Studies on the molecular and cellular characteristics are essential for the prognosis of patients with STAD. The recent developments in epigenetics has demonstrated that chromatin accessibilities are closely correlated with tumors. Chromatin accessibility is a hallmark of active DNA regulatory elements (22, 23) and when found in a gene’s promoter region, it can potentially be a transcription factor regulator. To date, few studies have focused on the association between differentially expressed genes, chromatin accessibilities, and tumor-infiltrating immune cells.

ATAC-seq is a novel experimental method, used to map open chromatin sites, predict transcription factor binding, and determine nucleosome positions. We acquired ATAC-seq datasets from TCGA to explore the genes with chromatin accessibilities in its promoter region. In other words, these genes may be potentially regulated by a transcription factor and have differential expression. By combining ATAC-seq and RNA-seq results, we identified a group of genes that were differentially expressed in STAD and possessed significant chromatin accessibilities. Of these genes, according to the analysis of TCGA datasets, the differentially expressed IL18BP might be a key molecule affecting STAD prognosis, validated by multiple databases including the GEO (GSE15459) and kmplot datasets. This suggested that IL18BP might have great potential to become a promising target for STAD treatment.

IL18BP, also known as interleukin 18 binding protein, is a secreted protein that is expressed in many cell types such as mononuclear and epithelial cells. IL18BP binds to IL18 and inhibits the binding of the latter to its receptor, leading to the inhibition of IL18-induced IFN-γ production (24). IL18BP has previously been shown to be differentially expressed and potentially correlated with the prognosis of patients with ovarian and colon cancers. However, it is unknown whether IL18BP influences the survival of patients with STAD and whether it plays a role in other pathways. Our findings have supported its prognostic value in patients with STAD.

We performed GSVA to quantize pathway expressions and to identify differentially expressed pathways in STAD. Interestingly, the immune-related pathways were the most relevant with IL18BP expression. The immune system plays an important role in tumor development. Thus, we hypothesized that IL18BP might influence STAD prognosis through the integration of tumor infiltrated immune cells.

To further explore the correlations between tumor infiltrated immune cells and IL18BP, we used the CIBERSORT algorithm for immune cell quantization. Based on the correlation analysis, we found that IL18BP was significantly associated with CD4+ T cells. IL18BP specifically binds to IL18 to neutralize its biological activity such as IFN-γ induction. As a natural secreted product, IL18BP could be a potential therapeutic target for diseases mediated in part by IFN-γ or IL18 itself. Interestingly, we demonstrated that IL18BP was differentially expressed in STAD tissues and significantly correlated with CD4+ T memory cells. As the CD4+ T cells were an important component of TILs and contributed to IFN-γ production, we hypothesized that the interactions between IL18BP and TILs, might play an important role in the occurrence and development of gastric adenocarcinoma, and further affect the prognosis of patients with STAD.

The prognostic predicting nomogram was constructed based on TILs. According to the reduced cox model, the expression level of the activated CD4+ T memory and T follicular helper cells and Tregs could evaluate the prognosis effectively. Previous studies have improved our understanding of the functions and origins of TILs and demonstrated that tumor-infiltrating immune cells are associated with the prognosis of STAD, especially in CD4+ and CD8+ T cells. Among varying subsets of TILs, the CD4+ T cells accounted for a great proportion and may play an important role in the tumor microenvironment. However, currently in human tissues, the molecular mechanisms of CD4+ TILs remain relatively elusive. The phenotypes and functions of memory CD4+ T cells remain poorly understood. We took both the IL18BP gene expression and the fraction of tumor-infiltrating immune cells into consideration. We inferred that the correlation between IL18BP and the immune infiltration of CD4+ T cells could be essential in STAD prognosis.

Interestingly, Aaron M. Ring et al. (25) proposed that IL18BP in the tumor microenvironment may act as a secreted immune checkpoint. Based on this, they constructed a decoy-resistant IL-18 and demonstrated its *in vivo* efficiency at treating the tumors. We highlighted the integration of IL18BP and CD4+ T cells, which could supplement this hypothesis. Through bioinformatics and clinical results, we inferred that IL18BP might be directly regulated by p53 and influence the STAD prognosis through the integration with CD4+ TILs. The integration of IL18BP and tumor-infiltrating CD4+ T cells could be a potential target for STAD therapy.

There are several inevitable limitations in this study that need to be addressed. First, the amount of public ATAC-seq data available is relatively limited. This may lead to potential bias in the parameters analyses. However, to minimize the bias, we used multidimensions verification to detect gene expressions and prognostic value of IL18BP. Second, this was a correlational study and did not investigate the biological mechanisms involved. Future studies should focus on experimentally verifying our findings. Despite the limitations mentioned above, we were the first to infer that the IL18BP expression might be regulated by chromatin accessibility and correlated with CD4+ T cells in gastric adenocarcinoma. We further verified this through qPCR and IHC. We also constructed the prognostic predicting nomograms for patients with STAD based on several public datasets.

Future studies should concentrate on delineating the direct molecular biological mechanisms of these correlations and investigate the intercellular communications between the gastric adenocarcinoma cells and CD4+ TILs. The integration of IL18BP and CD4+TILs could be a potential target for assessing STAD prognosis.

## Data Availability Statement

Publicly available datasets were analyzed in this study. This data can be found here: https://portal.gdc.cancer.gov/, https://xenabrowser.net/, and https://gdc.cancer.gov/about-data/publications/ATACseq-AWG.

## Ethics Statement

The studies involving human participants were reviewed and approved by Shanghai Tongji Hospital (reference number 2018-LCYJ-005). The patients/participants provided their written informed consent to participate in this study.

## Author Contributions

Conception/design: CH, RH, HC, ZN, QH, ZH, BG. Collection and/or assembly of data: CH, RH, HC, ZN, QH. Data analysis and interpretation: CH, RH, HC, ZN, QH, ZH, BG. Manuscript writing: CH, RH, QH, ZH, BG. Final approval of manuscript: CH, RH, HC, ZN, QH, ZH, BG. All authors contributed to the article and approved the submitted version.

## Funding

This study was funded by the Shanghai Science and Technology Innovation Action Plan (Grant No. 19441905700) and the Clinical Research and Cultivation Project of Shanghai Tongji Hospital (Grant No. ITJ (ZD) 1802, ITJ (ZD) 1804).

## Conflict of Interest

The authors declare that the research was conducted in the absence of any commercial or financial relationships that could be construed as a potential conflict of interest.

## Supplementary Material

Supplementary Figure 1 TP53 was closely related to IL18BP in STAD. **(A)** Pearson correlation analysis of the tumor-infiltrating immune cells, TP53 and IL18BP. **(B)** The expression of TP53 was highly correlated with IL18BP [r = 0.39 (Pearson), p < 0.001]. And the correlation between TP53 and activated CD4+ memory T cells was statistically significant but weak [r = 0.13 (Pearson), p = 0.01].
