# Supplementary figures and images for: Chromatin Accessibility Regulates Gene Expression and Correlates With Tumor-Infiltrating Immune Cells in Gastric Adenocarcinoma
Source: Front Oncol. 2021 Jan 5;10:609940. doi: 10.3389/fonc.2020.609940 (PMC7813815; doi:10.3389/fonc.2020.609940)

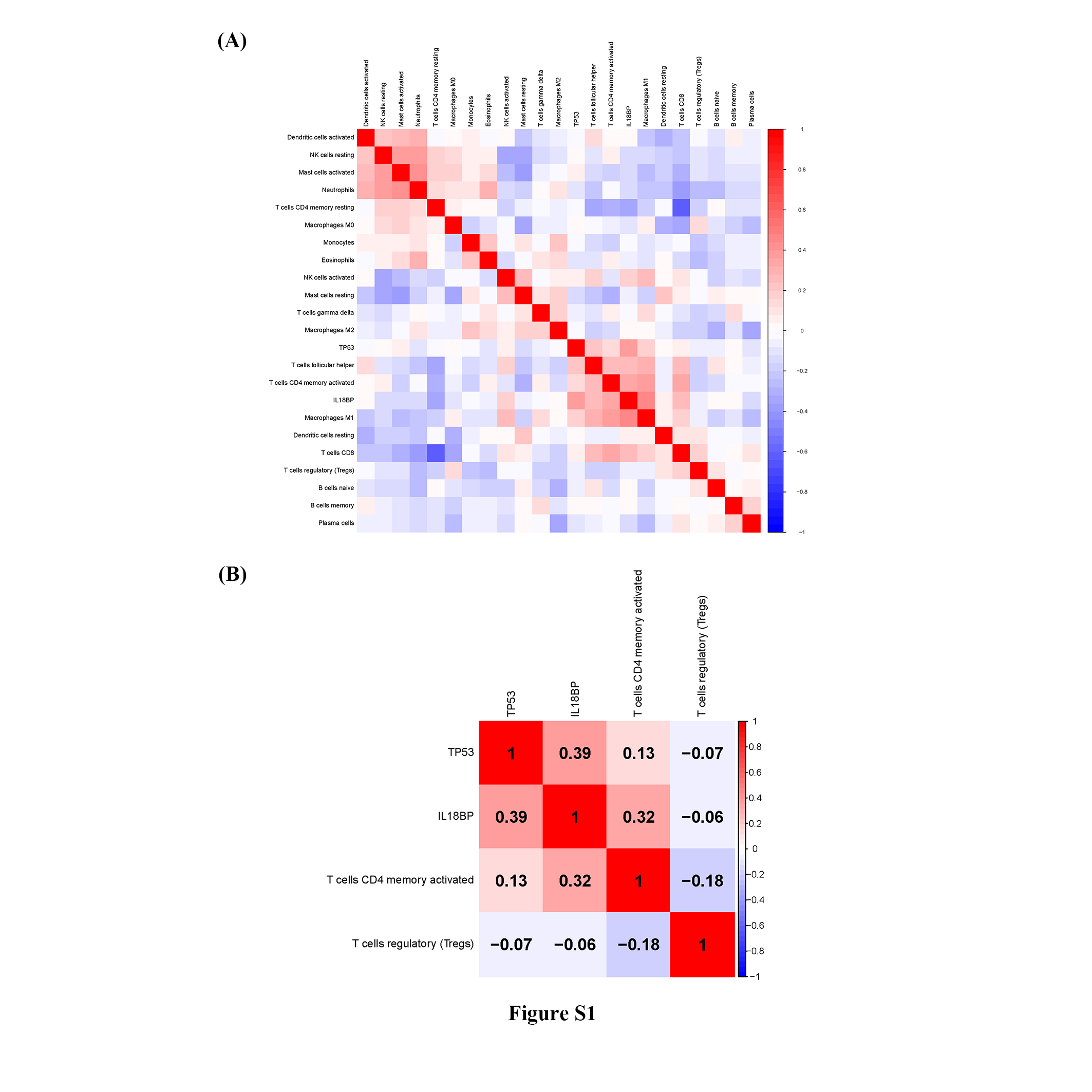

Supplement: Supplementary Figure 1 — TP53 was closely related to IL18BP in STAD. (A) Pearson correlation analysis of the tumor-infiltrating immune cells, TP53 and IL18BP. (B) The expression of TP53 was highly correlated with IL18BP [r = 0.39 (Pearson), p < 0.001]. And the correlation between TP53 and activated CD4+ memory T cells was statistically significant but weak [r = 0.13 (Pearson), p = 0.01]. [file Image_1.tif]
