# Supplementary material for: Chromatin Accessibility Regulates Gene Expression and Correlates With Tumor-Infiltrating Immune Cells in Gastric Adenocarcinoma
Source: Front Oncol. 2021 Jan 5;10:609940. doi: 10.3389/fonc.2020.609940 (PMC7813815; doi:10.3389/fonc.2020.609940)
Supplement: Supplementary file 2 [file Table_1.docx]

**Table S1** Baseline information of 321 patients diagnosed with stomach adenocarcinoma

| Variables | Total Patients (N = 321) |
| --- | --- |
| **Age, years** |  |
| Mean ± SD | 65.28 ± 10.36 |
| Median (Range) | 67 (35 - 88) |
| **Gender** |  |
| Female | 116 (36.14%) |
| Male | 205 (63.86%) |
| **Stage** |  |
| Stage I | 1 (0.31%) |
| Stage IA | 11 (3.43%) |
| Stage IB | 32 (9.97%) |
| Stage II | 25 (7.79%) |
| Stage IIA | 31 (9.66%) |
| Stage IIB | 48 (14.95%) |
| Stage III | 3 (0.93%) |
| Stage IIIA | 56 (17.45%) |
| Stage IIIB | 46 (14.33%) |
| Stage IIIC | 27 (8.41%) |
| Stage IV | 29 (9.03%) |
| unknow | 12 (3.74%) |
| **T** |  |
| T1 | 4 (1.25%) |
| T1a | 2 (0.62%) |
| T1b | 9 (2.80%) |
| T2 | 57 (17.76%) |
| T2a | 7 (2.18%) |
| T2b | 10 (3.12%) |
| T3 | 149 (46.42%) |
| T4 | 22 (6.85%) |
| T4a | 39 (12.15%) |
| T4b | 18 (5.61%) |
| TX | 4 (1.25%) |
| **N** |  |
| N0 | 94 (29.28%) |
| N1 | 91 (28.35%) |
| N2 | 65 (20.25%) |
| N3  N3a  N3b | 22 (6.85%)  36 (11.21%)  4 (1.25%) |
| NX | 8 (2.49%) |
| unknow | 1 (0.31%) |
| **M** |  |
| M0 | 292 (90.97%) |
| M1 | 19 (5.92%) |
| MX | 10 (3.12%) |

**Abbreviations:** SD, Standard deviation.
