# Supplementary material for: Chromatin Accessibility Regulates Gene Expression and Correlates With Tumor-Infiltrating Immune Cells in Gastric Adenocarcinoma
Source: Front Oncol. 2021 Jan 5;10:609940. doi: 10.3389/fonc.2020.609940 (PMC7813815; doi:10.3389/fonc.2020.609940)
Supplement: Supplementary file 3 [file Table_2.docx]

| seqnames | start | end | annotation | SYMBOL |
| --- | --- | --- | --- | --- |
| chr8 | 144795801 | 144796299 | Promoter (2-3kb) | ZNF517 |
| chr14 | 65098594 | 65099092 | Promoter (2-3kb) | MAX |
| chr16 | 84313044 | 84313542 | Promoter (2-3kb) | WFDC1 |
| chr1 | 112700145 | 112700643 | Promoter (1-2kb) | MOV10 |
| chr5 | 112417768 | 112418266 | Promoter (1-2kb) | EPB41L4A |
| chr1 | 93845733 | 93846231 | Promoter (<=1kb) | BCAR3 |
| chr2 | 177392487 | 177392985 | Promoter (<=1kb) | LOC100130691 |
| chr2 | 231000238 | 231000736 | Promoter (<=1kb) | SPATA3 |
| chr5 | 136192976 | 136193474 | Promoter (<=1kb) | SMIM32 |
| chr6 | 158907872 | 158908370 | Promoter (<=1kb) | C6orf99 |
| chr8 | 1816770 | 1817268 | Promoter (<=1kb) | MIR596 |
| chr9 | 33239891 | 33240389 | Promoter (<=1kb) | SPINK4 |
| chr9 | 137176476 | 137176974 | Promoter (<=1kb) | ANAPC2 |
| chr11 | 3580033 | 3580531 | Promoter (<=1kb) | LOC101927708 |
| chr11 | 71999325 | 71999823 | Promoter (<=1kb) | IL18BP |
| chr12 | 122944501 | 122944999 | Promoter (<=1kb) | ABCB9 |
| chr14 | 104108154 | 104108652 | Promoter (<=1kb) | ASPG |
| chr15 | 78092224 | 78092722 | Promoter (<=1kb) | SH2D7 |
| chr17 | 1684166 | 1684664 | Promoter (<=1kb) | PRPF8 |
| chr17 | 7404599 | 7405097 | Promoter (<=1kb) | NLGN2 |
| chr17 | 17590884 | 17591382 | Promoter (<=1kb) | PEMT |
| chr18 | 45839346 | 45839844 | Promoter (<=1kb) | SIGLEC15 |
| chr19 | 344608 | 345106 | Promoter (<=1kb) | MIER2 |
| chr19 | 7069133 | 7069631 | Promoter (<=1kb) | ZNF557 |
| chr19 | 49929810 | 49930308 | Promoter (<=1kb) | ATF5 |
| chr22 | 20956249 | 20956747 | Promoter (<=1kb) | LINC01637 |
| chrX | 30889230 | 30889728 | Promoter (<=1kb) | TAB3 |
| chrX | 48589695 | 48590193 | Promoter (<=1kb) | WDR13 |
| chr14 | 50018261 | 50018759 | Intron (uc284nlb.1/196913, intron 5 of 11) | LINC01599 |
| chr14 | 61472162 | 61472660 | Intron (uc059cfd.1/5583, intron 1 of 3) | PRKCH |
| chr12 | 124396877 | 124397375 | Intron (uc058uws.1/9612, intron 16 of 46) | NCOR2 |
| chr12 | 120394578 | 120395076 | Intron (uc058ube.1/uc058ube.1, intron 3 of 3) | MSI1 |
| chr2 | 235543343 | 235543841 | Intron (uc021vyp.1/116987, intron 1 of 9) | AGAP1 |
| chr9 | 129962962 | 129963460 | Intron (uc004byw.2/23048, intron 4 of 16) | FNBP1 |
| chr8 | 143873922 | 143874420 | Intron (uc003zaa.3/83481, intron 1 of 1) | EPPK1 |
| chr8 | 140153854 | 140154352 | Intron (uc003yvh.2/83696, intron 17 of 22) | TRAPPC9 |
| chr8 | 140159523 | 140160021 | Intron (uc003yvh.2/83696, intron 17 of 22) | TRAPPC9 |
| chr20 | 50824028 | 50824526 | Intron (uc002xvq.4/55653, intron 2 of 5) | BCAS4 |
| chr20 | 38874747 | 38875245 | Intron (uc002xje.3/26051, intron 2 of 10) | PPP1R16B |
| chr20 | 19947563 | 19948061 | Intron (uc002wro.3/54453, intron 3 of 11) | RIN2 |
| chr19 | 48351203 | 48351701 | Intron (uc002pix.2/55260, intron 3 of 7) | TMEM143 |
| chr18 | 12574751 | 12575249 | Intron (uc002kre.4/56907, intron 2 of 16) | SPIRE1 |
| chr1 | 66301089 | 66301587 | Intron (uc001dcn.4/5142, intron 7 of 16) | PDE4B |
| chr11 | 134762106 | 134762604 | Exon (uc058jja.1/uc058jja.1, exon 2 of 2) | LOC100507548 |
| chr17 | 4003746 | 4004244 | Downstream (<1kb) | ZZEF1 |
| chr1 | 89310476 | 89310974 | Distal Intergenic | GBP5 |
| chr6 | 157966172 | 157966670 | Distal Intergenic | SYNJ2 |
| chr6 | 167423935 | 167424433 | Distal Intergenic | TCP10 |
| chr8 | 38064099 | 38064597 | Distal Intergenic | EIF4EBP1 |
| chr8 | 94234317 | 94234815 | Distal Intergenic | CDH17 |
| chr11 | 9529200 | 9529698 | Distal Intergenic | ZNF143 |
| chr12 | 724357 | 724855 | Distal Intergenic | WNK1 |
| chr12 | 124716288 | 124716786 | Distal Intergenic | SCARB1 |
| chr13 | 48591894 | 48592392 | Distal Intergenic | LINC00462 |
| chr15 | 50798664 | 50799162 | Distal Intergenic | SPPL2A |
| chr11 | 72827989 | 72828487 | 3' UTR | ATG16L2 |
| chr19 | 38731121 | 38731619 | 3' UTR | ACTN4 |
